# Promotion of Survival and Engraftment of Transplanted Adipose Tissue-Derived Stromal and Vascular Cells by Overexpression of Manganese Superoxide Dismutase

**DOI:** 10.3390/ijms17071082

**Published:** 2016-07-07

**Authors:** Silvia Baldari, Giuliana Di Rocco, Angelo Trivisonno, Daniela Samengo, Giovambattista Pani, Gabriele Toietta

**Affiliations:** 1 Department of Research, Advanced Diagnostic, and Technological Innovation, Regina Elena National Cancer Institute, via E. Chianesi 53, Rome 00144, Italy; silvia.baldari@yahoo.it (S.B.); giuliana.dirocco@ifo.gov.it (G.D.R.); 2 Department of Surgical Science, Policlinico Umberto I, University of Rome “La Sapienza”, Viale Regina Elena 324, Rome 00161, Italy; angelo.trivisonno@uniroma1.it; 3 Institute of General Pathology, Laboratory of Cell Signaling, Università Cattolica School of Medicine, Largo F. Vito 1, Rome 00168, Italy; daniela.samengo@libero.it (D.S.); Giovambattista.Pani@unicatt.it (G.P.)

**Keywords:** adipose tissue-derived stromal and vascular cells, bioluminescence imaging, cell therapy, cell survival, cell transplantation, hypoxia, manganese superoxide dismutase, oxidative stress, reactive oxygen species

## Abstract

Short-term persistence of transplanted cells during early post-implant period limits clinical efficacy of cell therapy. Poor cell survival is mainly due to the harsh hypoxic microenvironment transplanted cells face at the site of implantation and to anoikis, driven by cell adhesion loss. We evaluated the hypothesis that viral-mediated expression of a gene conferring hypoxia resistance to cells before transplant could enhance survival of grafted cells in early stages after implant. We used adipose tissue as cell source because it consistently provides high yields of adipose-tissue-derived stromal and vascular cells (ASCs), suitable for regenerative purposes. Luciferase positive cells were transduced with lentiviral vectors expressing either green fluorescent protein as control or human manganese superoxide dismutase (*SOD2*). Cells were then exposed in vitro to hypoxic conditions, mimicking cell transplantation into an ischemic site. Cells overexpressing *SOD2* displayed survival rates significantly greater compared to mock transduced cells. Similar results were also obtained in vivo after implantation into syngeneic mice and assessment of cell engraftment by in vivo bioluminescent imaging. Taken together, these findings suggest that ex vivo gene transfer of *SOD2* into ASCs before implantation confers a cytoprotective effect leading to improved survival and engraftment rates, therefore enhancing cell therapy regenerative potential.

## 1. Introduction

Cell therapy is an innovative approach for the treatment of some acute and chronic degenerative conditions [1]. Transplanted cells may promote tissue regeneration through different mechanisms, including cell differentiation, cell fusion and paracrine effects via the secretion of various cytokines, growth factors and/or microvesicles. At any rate, the therapeutic effect of cell-based therapies ultimately depends on the persistence of cells at the site of tissue injury. Poor transplanted cell survival soon after in vivo transfer is slowing research progress and clinical translation of cell therapy [2]. One of the major reasons for early implanted cell death is the lack of oxygenation resulting for delayed revascularization at the transplantation site. Oxygen diffusion only sustains cells to a distance of approximately 200 micron from the oxygen source [3]. Thus, direct injection into damaged tissue exposes transplanted cells to prolonged periods of hypoxia. In addition, cell transplantation procedures involve cell detachment from the extracellular matrix; disruption of cell-matrix contact also promotes a strong increase of reactive oxygen species (ROS) production, triggering anoikis [4]. Therefore, the benefits of cell transplantation could be improved by adapting donor cells in order to enhance their resistance to hypoxic stress [5,6,7,8].

Anti-death strategies have been explored to promote the survival of transplanted cells and increase the therapeutic potential of transplantation therapy [9,10,11]. These methods include continuous infusion of trophic factors and hypoxic preconditioning [12]. Alternatively, survival and regenerative capability of transplanted cells can be improved in vitro before transplantation [13,14,15,16].

Response to oxidative stress and anoikis are very complex and the networks of genes involved not fully elucidated. Cell exposure to low oxygen leads to an increased generation of oxygen species by dysfunctional mitochondria [17]. Accordingly, the mitochondrial superoxide scavenger superoxide dismutase 2 (*MnSOD* or *SOD2*) gene is strongly upregulated during hypoxic preconditioning, underlining its pivotal role in hypoxic stress resistance by ROS detoxification [18]. In fact, *SOD2* helps in preventing peroxynitrite formation by conversion of superoxide anion to hydrogen peroxide, which is then reduced to water. Moreover, *SOD2* expression in highly upregulated during cell detachment from the extracellular matrix in order to confer anoikis resistance [19].

Adipose tissue represents an attractive source for isolating stromal and vascular cells suitable for cell therapies aimed at tissue regeneration [20,21,22]. Adipose tissue-derived stromal cells (ASCs) [23] are collected from adipose tissue by collagenase digestion and differential centrifugation. ASCs are able to differentiate into several cells types of both mesodermal and nonmesodermal origin, including adipocytes, chondrocytes, osteocytes, myocytes, hepatocytes, endocrine pancreatic cells, and neurons [21]. In particular, ASCs are suitable for vascular regeneration, since they are able to differentiate into endothelial cells and form vessel-like structures that assume endothelial function in Matrigel [24,25,26]. Moreover, ASCs are able to exert an antioxidant effect via a paracrine mechanism [27].

Here we provide evidence that ex vivo genetic modification of ASCs by lentiviral-mediated *SOD2* gene transfer provides a benefit by promoting cell survival to hypoxia in vitro and by enhancing engraftment in vivo.

## 2. Results

### 2.1. Lentiviral-Mediated Gene Transfer into Adipose-Tissue-Derived Stromal and Vascular Cells (ASCs)

**Figure 1:**
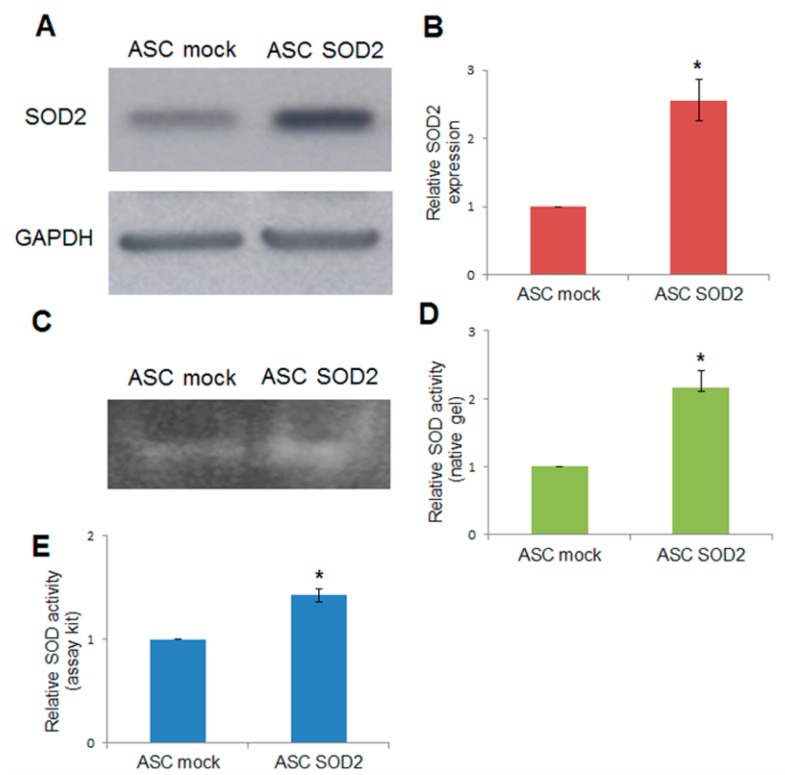
Lentiviral-mediated *SOD2* gene transfer into ASCs. (**A**) Immunoblotting and (**B**) densitometric analysis in mock- and *SOD2*-transduced ASCs: the blot was probed with a primary antibody specific for human *SOD2*, and one directed against GAPDH as normalization control. SOD-activity determined by (**C**) non-denaturing polyacrylamide gel electrophoresis and riboflavin-nitroblue tetrazolium staining followed by (**D**) densitometric analysis and (**E**) SOD determination kit in mock- and *SOD2*-transduced ASCs. In each panel, the asterisk (*) indicates a significant difference versus the control group, assessed by unpaired Mann–Whitney *U* test (*p* < 0.05).

Adipose tissue-derived stromal and vascular cells were isolated from lipoaspirates obtained from human donors [24]. A lentiviral vector (LV) for expression of human superoxide dismutase 2 (*SOD2*) was generated and used for gene transfer into ASCs. Immunoblotting using anti-*SOD2* antibodies followed by densitometric analysis revealed a 2.5-fold increased expression upon *SOD2* gene transfer, compared to mock-transduced ASCs (Figure 1A,B). In addition, SOD activity was assayed by non-denaturing polyacrylamide gel electrophoresis followed by specific riboflavin-nitroblue tetrazolium staining and densitometric analysis (Figure 1C,D). Further determination of SOD activity after lentiviral-mediated *SOD2* gene transfer into ASCs was obtained using a specific determination kit (Sigma-Aldrich, St. Louis, MO, USA) (Figure 1E). Both methods were in agreement in determining that enzymatic activity in *SOD2* overexpressing cells was approximately 1.5–2.5 folds higher compared to mock-transduced cells.

We aimed at testing whether *SOD2* overexpression into ASCs might result into enhanced cell engraftment rate in vivo upon transplant into an experimental animal model. A suitable way to assess improved engraftment of *SOD2*-expressing ASC, in comparison with mock transduced ASC, is by means of sensitive, noninvasive bioluminescent imaging (BLI) upon firefly luciferase gene transfer. To this end, we needed to obtain murine ASCs expressing both firefly luciferase and *SOD2*. We therefore tested the susceptibility of freshly isolated murine ASCs to lentiviral-mediated gene transfer in suspension, using a “Suspension with Lentiviral vectors and Immediately Transplanted (SLIT)” protocol originally developed for gene transfer into transplantable primary hepatocytes [28]. Extensive ex vivo expansion may affect chromosomal stability, differentiation potential and cell adhesion characteristic of ASCs, possibly modifying their homing capabilities [29]. The described methodology allows for high rates gene transfer through minimal in vitro manipulation, therefore reducing the issues associated with prolonged in vitro long-term cell expansion. We used a LV expressing enhanced green fluorescent protein (GFP) under the ubiquitous human phosphoglycerate kinase (PGK) promoter [30]. Upon lentiviral-mediated gene transfer at multiplicity of infection 30, in suspension for two hours we achieve up to 89% transduction, as assessed by GFP expression determined by flow cytometric (FACS) and fluorescent microscopy analysis performed after 48 h of culture in order to allow for efficient transgene expression. Using this procedure, we obtained firefly luciferase (fLuc) expressing ASCs, which were co-transduced with LV vectors expressing either GFP as control or *SOD2*. Luciferase-positive cells expressing either GFP or *SOD2* had a comparable light emission profile as assessed by BLI imaging indicating that co-expression with a second transgene does not affect luciferase expression.

### 2.2. Lentiviral-Mediated Gene Transfer into ASCs of Superoxide Dismutase 2 (SOD2) Confers Improved Resistance to Hypoxia in Vitro

**Figure 2:**
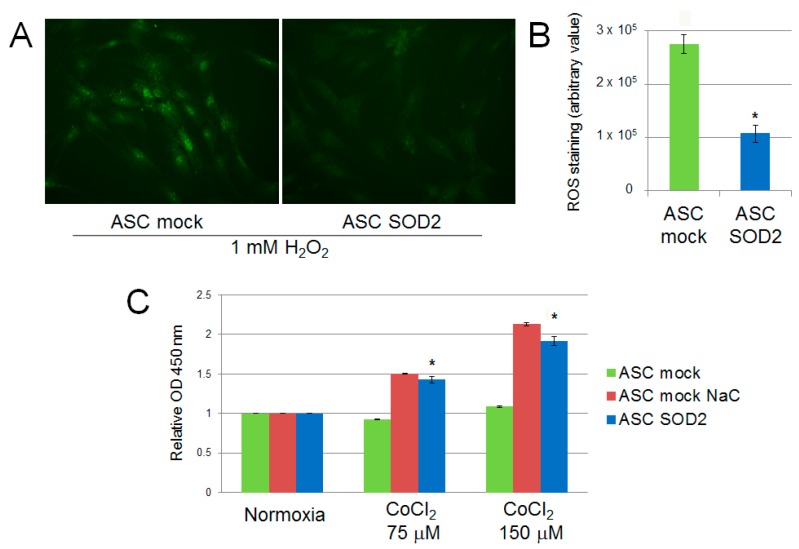
ROS production in *SOD2* overexpressing human ASC after acute hypoxic challenge (1 mM H_2_O_2_ for 30 min) assessed using (**A**) the CellROX oxidative stress fluorogenic probe (original magnification 40×) and (**B**) fluorescence quantification with ImageJ software. Cell viability assay after 24 h of culture in cobalt chloride-induced hypoxic conditions (**C**). Treatment with the antioxidant scavenger N-acetylcysteine (NaC) (2 mM) was used as control. Results are reported as mean ± S.E.M. of three independent experiments. Significance was assessed by Mann–Whitney *U* test; * *p* < 0.05 versus ASC mock.

We evaluated the performance of mock- and *SOD2*-transduced human ASCs cultured in hypoxic condition in vitro. Firstly, we assessed the production of ROS following exposure of ASCs to 1 mM H_2_O_2_ for 30 min. *SOD2*-expressing cell culture displayed reduced ROS production following acute hypoxic stress, as determined by fluorescence microscopy with the ROS-sensitive CellROX oxidative stress reagent fluorogenic probe (Figure 2A). Additionally, ASCs were exposed to prolonged hypoxic conditions achieved by adding increasing concentration of CoCl_2_ to the culture medium. After 24 h cell viability was assessed by the WST-1 cell proliferation assay kit, reveling that *SOD2* expressing ASC viability was increased compared to mock ASC (*p* ≤ 0.05) and comparable to the levels of ASC mock cells treated with the antioxidant N-acetylcysteine (NaC) (Figure 2B).

**Figure 3:**
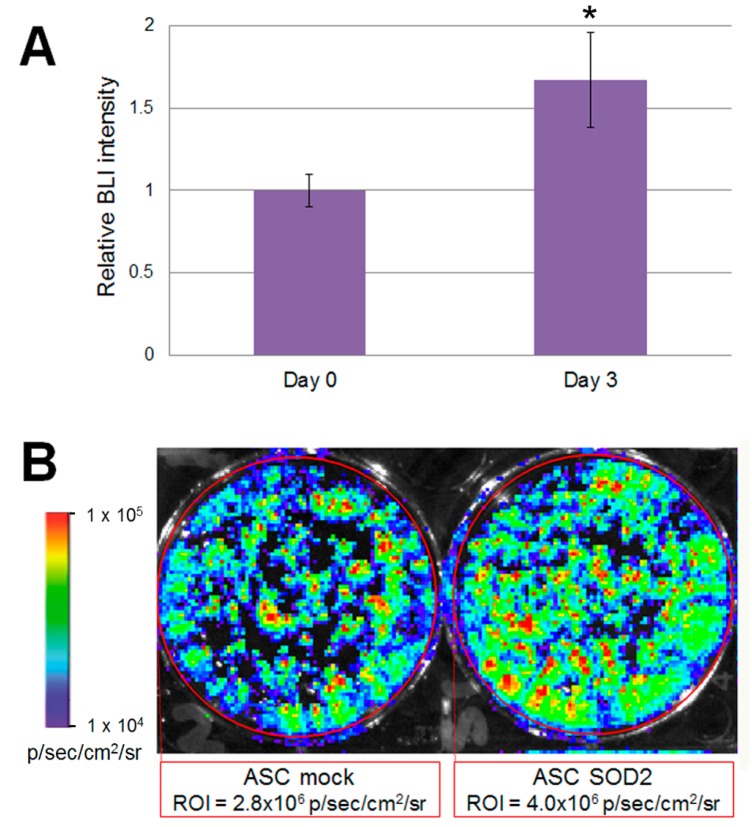
Luciferase positive *SOD2*-expressing ASCs display improved ability to withstand hypoxia in vitro. Murine ASCs expressing luciferase were transduced with LV vectors expressing either GFP as mock control or *SOD2* and cultured in hypoxic conditions. (**A**) BLI analysis was performed at three days of culture and intensity of bioluminescence was determined. Data are expressed as the ratio of the BLI signal of the *SOD2* group vs. mock control group. Data are expressed as mean ± SEM of three different independent experiments performed in duplicate. The asterisk indicates a significant difference determined by unpaired Mann–Whitney *U* test (*p* < 0.05); (**B**) A representative BLI analysis and quantification of ASCs cultured in hypoxic condition for three days.

Accordingly, to validate our experimental design before studies involving animals, we wanted to test the performance of mock- and *SOD2*-transduced, luciferase expressing murine ASCs cultured in hypoxic condition in vitro. To mimic the hypoxic environment which cell face upon transplantation, we cultured luciferase-expressing ASCs in a modular airtight humidified chamber flushed with a gas mixture which consisted of 95% N_2_ and 5% CO_2_ for three days. Bioluminescent imaging (BLI) analysis was performed before hypoxia induction and after three days of culture in hypoxic condition (Figure 3). At the 72 h time point, BLI imaging signal detected in *SOD2*-overexpressing cells was statistically significantly higher than the one observed in mock transduced cells (Figure 3B). In the current experimental setting, stronger bioluminescence signal correlates with improved survival of *SOD2*-expressing cells, compared to mock (GFP)-transduced control. BLI analysis performed on cells undergoing the same gene transfer procedure, but maintained in normoxic condition did not reveal any significant difference amongst the groups.

### 2.3. ASCs Expressing SOD2 Have an Improved Engraftment Rate into a Matrigel Plug in Vivo

**Figure 4:**
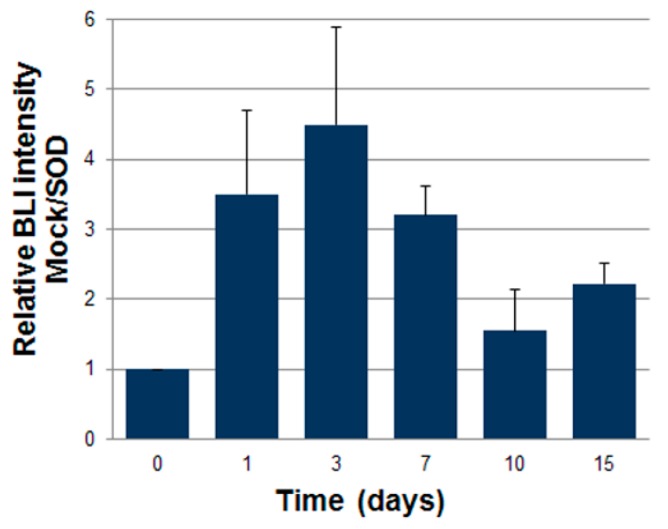
*SOD2*-expressing ASCs show improved engraftment potential in vivo. Adipose tissue-derived stromal and vascular cells (ASCs) expressing luciferase after lentiviral-mediated gene transfer of either GFP (mock) or superoxide dismutase (*SOD2*) cells were injected subcutaneously (7 × 10^5^ cells/mouse) into a Matrigel plug into syngeneic mice (*n* = 6 per group). At different time points, bioluminescence imaging analysis (BLI) was performed and quantified. Data are expressed as the ratio of the BLI signal of the *SOD2* group vs. mock control group. Difference of BLI signals between control (GFP) and *SOD2* group was significant at all analyzed time points.

**Figure 5:**
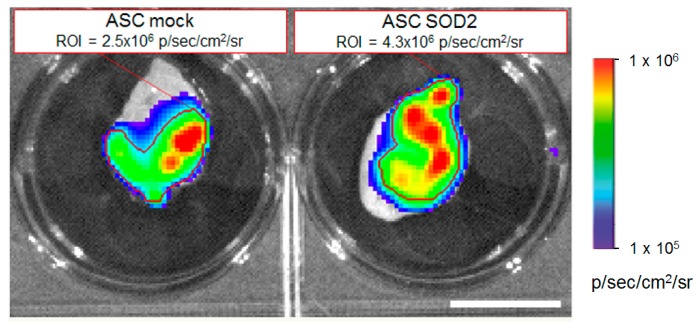
*SOD2*-expressing ASCs show improved engraftment potential: ex vivo gel plug BLI analysis. Analysis of gel plugs excised at necropsy from one representative animal per group three days after implantation of Cultrex containing 7 × 10^5^ luciferase-positive ASCs expressing either GFP (mock control) or *SOD2*. Quantification of the BLI signals in the respective selected regions of interest (indicated by the red lines) confirmed improved engraftment of *SOD2*-expressing cells compared to mock control. Scale bar: 1 cm.

Luciferase expressing ASCs were transduced with LV vectors expressing either GFP as control or *SOD2*, as described above. Then cells were mixed with Cultrex and a plug containing 7 × 10^5^ cells was administered subcutaneously into syngeneic mice. We followed engraftment of ASCs by BLI performed at different time points after gel plug implant (Figure 4). The intensity of bioluminescence in the group of animals receiving *SOD2*-expressing ASCs was consistently higher than in the group receiving mock transduced cells, suggesting a role of *SOD2* in promoting ASCs engraftment. At three days after implantation, selected animals were sacrificed, gel plugs were excised and BLI imaging performed (Figure 5). Quantification of the bioluminescence signal from excised gel plugs confirmed improved engraftment of cells expressing *SOD2*, compared to mock transduced cells (Figure 5).

## 3. Discussion

Finding new avenues to promote cell survival of transplanted cells would represent an important strategic advancement to the clinical success of cell therapy for regenerative medicine. Upon administration, transplanted cells encounter severe conditions, characterized by poor blood supply, low nutrients and excess of oxygen species (ROS), which initiate a cascade of events that leads to apoptotic cell death, preventing cell survival in the early stages after transplant [31]. Therefore, early survival of transplanted cells ultimately dictates their possibility of engraftment and, in the end, their therapeutic potential. In fact, even if it is not possible to rule out a possible beneficial effect triggered by nonviable cells [32], persistence of transplanted cells is critical for the success of cell-based therapies for tissue regeneration.

Oxidative stress is characterized by an imbalance between the pro-oxidant and anti-oxidant stimuli leading to lipid peroxidation and protein and DNA damages. To cope with increased ROS levels, cells up-regulate the expression of antioxidant enzymes such as superoxide dismutase and catalase, which limit the levels of superoxide and hydrogen peroxide, respectively. In particular, modulation of *SOD2* has been observed in adipose tissue of obese patients, possibly to mitigate mitochondrial dysfunction correlated with the pathophysiology of obesity [33]. Accordingly, transplantation of genetically modified *SOD2*-overexpressing mesenchymal cells has been suggested as a new therapeutic approach for obesity-associated metabolic syndrome [34]. Moreover, *SOD2* has been identified as one of the genes mediating the late phase of ischemic preconditioning, defined as an increased tolerance to ischemia and reperfusion induced by a previous sub-lethal period of ischemia [35]. Hypoxic preconditioning of stem cells has been associated with improved regenerative abilities and enhanced in vivo homing to the site of injury [5,12,36]. Moderate hypoxic preconditioning has also been associated with an increase of ASCs proangiogenic properties [37]. The cytoprotective effects of preconditioning can also be achieved by pharmacological treatment, heat shock intervention and gene transfer to promote expression of pro survival genes [11]. Unfortunately, development of SODs as pharmaceutical products has been hampered by unfavorable pharmacologic and pharmacokinetic properties of the enzymes [38,39]. To counteract SOD rapid renal clearance, gene therapy approaches have been proposed to achieve robust SOD expression and enhanced recovery of tissue injuries has been described [40].

We investigated the possibility of improving survival of transplanted cells against excessive ROS production by *SOD2* ex vivo gene transfer. Hypoxia, due to poor vascularization, and anoikis, triggered by the lack of connection of cells with the extracellular matrix, are considered the main mechanisms responsible for reduced survival of transplanted cells. Both hypoxic stress and ECM detachment promotes ROS excess. SOD actively dismutates superoxide anions to hydrogen peroxide and molecular oxygen, thus neutralizing the oxygen radicals. Consequently, SOD overexpression is instrumental both to protect cells from oxidative stress and anoikis [19].

For precisely determining cells’ engraftment, persistence, and biodistribution after in vivo administration, we took advantage of the sensitive in vivo bioluminescence imaging technique (BLI). The procedure is non-invasive and facilitates repetitive imaging, thereby providing unprecedented insight to monitor on the same animal over time transplanted cell survival, proliferation and migration. Limitations of the procedure are associated to the fact that to produce a bioluminescence signal, which can be detected, transplanted luciferase-expressing cells should uptake and convert luciferin. Poor blood supply in the area of cell implantation may limit diffusion of luciferase administered by intraperitoneal administration. Conversely, during time, the formation of a vascular network into the Matrigel plug by ASCs may facilitate exposure of transplanted cells to luciferase, therefore leading to an increment of BLI signal. Another factor that should be taken into account in our experimental design is that the light-producing enzymatic conversion of luciferin to oxyluciferin requires ATP and oxygen [41]. Therefore, only living cells expressing luciferase will be able to produce a BLI signal; in other words, BLI intensity correlates with the number cells surviving the transplantation procedure. On the other hand, in the absence of an adequate oxygen supply, luciferase-expressing cells may not be able to fully convert luciferin, leading to an underestimation of the number of engrafted cells in a hypoxic environment.

In summary, we describe a gene therapy approach aimed at increasing the regenerative efficacy of cell therapy by conferring to the transplanted cells improved oxidative stress resistance. Notably, these results are in accordance with previous reports associating viral mediated overexpression of SOD with improved survival of transplanted pancreatic islet [42] and wound healing mediated be endothelial progenitor cells administration [43].

## 4. Experimental Section

### 4.1. Experimental Animals

We used adult FVB wild-type mice from colonies maintained in our institutional animal facility. All experimental procedures conformed to protocols approved by the Regina Elena National Cancer Institute Animal Care and Use Committee, and by the General Directorate for Animal Health and Veterinary Medicinal Products of the Italian Ministry of Health (Authorization n° 1001/2015-PR, 22 September 2015), according to the current National Legislation (Art. 31 D.lgs 26/2014, 4 March 2014).

### 4.2. Cells Isolation and Culture

Human ASC were isolated from lipoaspirates collected from the thigh and hip regions of 6 healthy, non obese (Body Mass Index below 30 kg/m^2^), Caucasian females (age range 25–56 years) undergoing liposuction, as previously described [24]. All donors gave their written informed consent in accordance with the standards of the University of Rome “La Sapienza” Ethical Committee (Authorization n° 1794/15, 13 February 2015) and the principles expressed in the Declaration of Helsinki. Murine adipose tissue-derived stromal cells (ASCs) [23] were isolated from 6–8 week-old FVB mice as previously described [44]. Briefly, inguinal subcutaneous fat pads were digested for 45 min in a shaking water bath at 37 °C in phosphate buffered saline (PBS) containing 2% bovine serum albumin (BSA) and 2 mg/mL collagenase A (Roche Diagnostics, Mannheim, Germany). Tissue debris were eliminated by filtration through a 40 µm cell strainer (BD Falcon, Franklin Lakes, NJ, USA) and cells collected by centrifugation (500× *g*). Cells were then washed in PBS, counted and used either for gene transfer procedures or culture. For cell culture cells were plated at 2.5 × 10^4^ cells/cm^2^ in α-MEM supplemented with 20% FBS, 2 mM l-glutamine, 1% penicillin-streptomycin in incubator at 37 °C and 5% CO_2_. The next day non-adherent cells were discarded, medium replaced and adipose-derived adherent stromal cells were allowed to grow until they reached 80% confluence.

We used different methods for induction of hypoxia in cell culture [45]. In some experiments, hypoxia was induced by placing cell culture dishes in airtight modular incubator chambers (Forma Scientific, Mountain View, CA, USA), flushed for 20 min with 95% N_2_ plus 5% CO_2_. In these conditions, oxygen concentration is approximately 1% [46]. Chambers were then sealed and placed in an incubator at 37 °C for the duration of the experiments. Alternatively, ASCs were incubated for 24 h in culture medium supplemented with CoCl_2_ (Sigma-Aldrich, St. Louis, MO, USA). Oxidative stress induction was also achieved by adding 1 mM H_2_O_2_ to ASC culture medium for 30 min. The CellROX Oxidative Stress Reagents fluorogenic probe (Thermo Fisher Scientific, Waltham, MA, USA) was used to measure reactive oxygen species (ROS) in live cells according to manufacturer’s specifications. This cell-permeable dye is weakly fluorescent in reduced state while in the presence of ROS it is converted to the oxidized form which emits a fluorescence signal directly proportional to oxidation and thus to the amount of ROS. Quantification of green fluorescence was performed using the ImageJ software (National Institutes of Health, Bethesda, MD, USA), as previously described [16]. Cell proliferation was measured using the WST-1 cell proliferation assay kit (Takara, Clontech, Mountain View, CA, USA), according to manufacturer’s instructions.

### 4.3. Lentiviral Vectors Production

We generated a third-generation self-inactivating lentiviral vector (LV) expressing human *SOD2* by cloning the full-length cDNA for *SOD2* derived from pcDNA3*SOD2* [47], as replacements to the E-GFP cDNA into the pCCLsin.cPPT.hPGK.E-GFP.Wpre (phosphoglycerate kinase promoter–enhanced jellyfish green fluorescent protein) plasmid [30], generously provided by Elisa Vigna (IRCC, Candiolo, Italy). Generation of a lentiviral vector expressing firefly luciferase has been previously described [48]. Recombinant vesicular stomatitis virus–pseudotyped LVs were obtained according to published protocols [49]. Titer of GFP-expressing LV stocks used as control was determined by serial dilution on HeLa cells and flow cytometry analysis and was above 1 × 10^8^ transducing units/mL (TU/mL).

### 4.4. Lentiviral-Mediated Gene Transfer into ASCs

Freshly isolated ASCs were transduced with a procedure named SLIT for “Suspension with Lentiviral vectors and Immediately Transplanted”, originally described for efficient lentiviral-mediated gene transfer into primary hepatocytes [28]. Briefly, uncultured ASCs were resuspended at a cell density of 5 × 10^5^ cells/mL in saline solution and exposed to lentiviral vectors at a multiplicity of infection of 30 for 2 h, with occasional shaking. Cells were then carefully washed and cultured as described above.

### 4.5. Immunoblotting and Activity Analysis

Expression of human *SOD2* after lentiviral-mediated gene transfer was assessed by Western blot analysis using primary antibodies against *SOD2* (1:1000) (Upstate Biotechnology–Millipore, Temecula, CA, USA), according to established protocols [50]. Densitometry analysis was performed using the ImageJ software (National Institutes of Health, Bethesda, MD, USA) to normalize the signals against glyceraldehyde phosphate dehydrogenase (GAPDH), considered as a loading control. SOD activity was assayed by non-denaturing polyacrylamide gel electrophoresis and staining with nitro blue tetrazolium, as previously described [50]. SOD activity was further assessed using the SOD determination kit (Sigma-Aldrich, St. Louis, MO, USA) according to the manufacturer’s instructions.

### 4.6. In Vivo Gel Plug Assay

The assay is based on the implant of Matrigel^®^ plugs and was performed as previously described [24]. Briefly, ASCs were resuspended in PBS and mixed with Cultrex^®^ growth factor reduced basement membrane extract (Trevigen Inc., Gaithersburgh, MD, USA). An aliquot of 400 µL of Cultrex^®^ containing 7 × 10^5^ cells was therefore injected subcutaneously into syngeneic mice.

### 4.7. Ex Vivo and in Vivo Optical Bioluminescent Imaging

Bioluminescent imaging (BLI) analysis was performed using the IVIS^®^ Lumina II equipped with the Living Image^®^ software for data quantification (PerkinElmer, Waltham, MA, USA). For ex vivo imaging, ASCs and gel plug explants containing luciferase expressing cells were placed into clear bottom tissue culture dishes and incubated in a saline solution containing d-luciferin (PerkinElmer) (150 µg/mL) before analysis. For in vivo analysis, mice were anesthetized with Avertin^®^ (2,2,2-tribromoethanol; Sigma-Aldrich, St. Louis, MO, USA) (240 mg/kg) and d-luciferin dissolved in PBS (150 mg/kg body weight) was administered i.p. 10 min before analysis [48]. Photons emitted from luciferase-expressing ASCs transplanted into the animals were collected with final accumulation times of 1 to 5 min, depending on the intensity of the bioluminescence emission.

### 4.8. Statistical Analysis

Results are expressed as means ± standard error of the mean (SEM). Data analysis and comparisons between control and treated groups were performed and the significance of differences was assessed with Mann–Whitney *U* test for unpaired data; statistical significance level was set at *p* ≤ 0.05.

## 5. Conclusion

Ex vivo gene transfer of human manganese superoxide dismutase into adipose tissue-derived stromal cells before implantation confers a cytoprotective effect leading to improved survival to hypoxia in vitro and increased engraftment rates in vivo. Therefore, cell preconditioning mediated by *SOD2* gene transfer might constitute a suitable method to increase the efficacy of cell-based therapies to promote tissue regeneration.

## References

[1] Dimmeler S., Ding S., Rando T.A., Trounson A. (2014). Translational strategies and challenges in regenerative medicine. Nat. Med..

[2] Menasche P. (2011). Cardiac cell therapy: Lessons from clinical trials. J. Mol. Cell. Cardiol..

[3] Tilkorn D.J., Bedogni A., Keramidaris E., Han X., Palmer J.A., Dingle A.M., Cowling B.S., Williams M.D., McKay S.M., Pepe L. (2010). Implanted myoblast survival is dependent on the degree of vascularization in a novel delayed implantation/prevascularization tissue engineering model. Tissue Eng. Part A.

[4] Song H., Cha M.J., Song B.W., Kim I.K., Chang W., Lim S., Choi E.J., Ham O., Lee S.Y., Chung N. (2010). Reactive oxygen species inhibit adhesion of mesenchymal stem cells implanted into ischemic myocardium via interference of focal adhesion complex. Stem Cells.

[5] Haider H.K.H., Ashraf M. (2010). Preconditioning and stem cell survival. J. Cardiovasc. Transl. Res..

[6] Chang W., Song B.W., Moon J.Y., Cha M.J., Ham O., Lee S.Y., Choi E., Hwang K.C. (2013). Anti-death strategies against oxidative stress in grafted mesenchymal stem cells. Histol. Histopathol..

[7] Sart S., Ma T., Li Y. (2014). Preconditioning stem cells for in vivo delivery. Biores. Open Access.

[8] Yang M., Xiao J., Liu Y. (2015). Endogenous antioxidant level of stem cell is important for the transplantation efficacy. Inflamm. Cell Signal..

[9] Ogle M.E., Yu S.P., Wei L. (2009). Primed for lethal battle: A step forward to enhance the efficacy and efficiency of stem cell transplantation therapy. J. Thorac. Cardiovasc. Surg..

[10] Penn M.S., Mangi A.A. (2008). Genetic enhancement of stem cell engraftment, survival, and efficacy. Circ. Res..

[11] Haider H.K.H., Ashraf M. (2008). Strategies to promote donor cell survival: Combining preconditioning approach with stem cell transplantation. J. Mol. Cell. Cardiol..

[12] Beegle J., Lakatos K., Kalomoiris S., Stewart H., Isseroff R.R., Nolta J.A., Fierro F.A. (2015). Hypoxic preconditioning of mesenchymal stromal cells induces metabolic changes, enhances survival and promotes cell retention in vivo. Stem Cells.

[13] Muscari C., Giordano E., Bonafè F., Govoni M., Pasini A., Guarnieri C. (2013). Priming adult stem cells by hypoxic pretreatments for applications in regenerative medicine. J. Biomed. Sci..

[14] Amiri F., Jahanian-Najafabadi A., Roudkenar M.H. (2015). In vitro augmentation of mesenchymal stem cells viability in stressful microenvironments: In vitro augmentation of mesenchymal stem cells viability. Cell Stress Chaperones.

[15] Lee S., Choi E., Cha M.J., Hwang K.C. (2015). Cell adhesion and long-term survival of transplanted mesenchymal stem cells: A prerequisite for cell therapy. Oxid. Med. Cell. Longev..

[16] Zeng W., Xiao J., Zheng G., Xing F., Tipoe G.L., Wang X., He C., Chen Z.Y., Liu Y. (2015). Antioxidant treatment enhances human mesenchymal stem cell anti-stress ability and therapeutic efficacy in an acute liver failure model. Sci. Rep..

[17] Guzy R.D., Hoyos B., Robin E., Chen H., Liu L., Mansfield K.D., Simon M.C., Hammerling U., Schumacker P.T. (2005). Mitochondrial complex III is required for hypoxia-induced ROS production and cellular oxygen sensing. Cell Metab..

[18] Chen W., Qiu J.F., Zhang Z.Q., Luo H.F., Rosello-Catafau J., Wu Z.Y. (2006). Gene expression changes after hypoxic preconditioning in rat hepatocytes. Hepatobiliary Pancreat. Dis. Int..

[19] Kamarajugadda S., Cai Q., Chen H., Nayak S., Zhu J., He M., Jin Y., Zhang Y., Ai L., Martin S.S. (2013). Manganese superoxide dismutase promotes anoikis resistance and tumor metastasis. Cell Death Dis..

[20] Gimble J.M., Bunnell B.A., Guilak F. (2012). Human adipose-derived cells: An update on the transition to clinical translation. Regen. Med..

[21] Zuk P.A. (2013). Adipose-derived stem cells in tissue regeneration: A review. ISRN Stem Cells.

[22] Trivisonno A., Abecassis M., Monti M., Toietta G., Bachir A., Shiffman M.A., di Giuseppe A., Bassetto F. (2014). Adipose tissue: From energy reservoir to a source of cells for epithelial tissue engineering. Stem Cells in Aesthetic Procedures.

[23] Bourin P., Bunnell B.A., Casteilla L., Dominici M., Katz A.J., March K.L., Redl H., Rubin J.P., Yoshimura K., Gimble J.M. (2013). Stromal cells from the adipose tissue-derived stromal vascular fraction and culture expanded adipose tissue-derived stromal/stem cells: A joint statement of the International Federation for Adipose Therapeutics and Science (IFATS) and the International Society for Cellular Therapy (ISCT). Cytotherapy.

[24] Trivisonno A., Di Rocco G., Cannistra C., Finocchi V., Farr S., Monti M., Toietta G. (2014). Harvest of superficial layers of fat with a microcannula and isolation of adipose tissue-derived stromal and vascular cells. Aesthet. Surg. J..

[25] Miranville A., Heeschen C., Sengenès C., Curat C.A., Busse R., Bouloumié A. (2004). Improvement of postnatal neovascularization by human adipose tissue-derived stem cells. Circulation.

[26] Cao Y., Sun Z., Liao L., Meng Y., Han Q., Zhao R.C. (2005). Human adipose tissue-derived stem cells differentiate into endothelial cells in vitro and improve postnatal neovascularization in vivo. Biochem. Biophys. Res. Commun..

[27] Kim W.S., Park B.S., Kim H.K., Park J.S., Kim K.J., Choi J.S., Chung S.J., Kim D.D., Sung J.H. (2008). Evidence supporting antioxidant action of adipose-derived stem cells: Protection of human dermal fibroblasts from oxidative stress. J. Dermatol. Sci..

[28] Nguyen T.H., Khakhoulina T., Simmons A., Morel P., Trono D. (2005). A simple and highly effective method for the stable transduction of uncultured porcine hepatocytes using lentiviral vector. Cell Transplant..

[29] Eggenhofer E., Luk F., Dahlke M.H., Hoogduijn M.J. (2014). The life and fate of mesenchymal stem cells. Front. Immunol..

[30] Follenzi A., Ailles L.E., Bakovic S., Geuna M., Naldini L. (2000). Gene transfer by lentiviral vectors is limited by nuclear translocation and rescued by HIV-1 *pol* sequences. Nat. Genet..

[31] Agrawal H., Shang H., Sattah A.P., Yang N., Peirce S.M., Katz A.J. (2014). Human adipose-derived stromal/stem cells demonstrate short-lived persistence after implantation in both an immunocompetent and an immunocompromised murine model. Stem Cell Res. Ther..

[32] Lu W., Fu C., Song L., Yao Y., Zhang X., Chen Z., Li Y., Ma G., Shen C. (2013). Exposure to supernatants of macrophages that phagocytized dead mesenchymal stem cells improves hypoxic cardiomyocytes survival. Int. J. Cardiol..

[33] Savini I., Catani M.V., Evangelista D., Gasperi V., Avigliano L. (2013). Obesity-associated oxidative stress: Strategies finalized to improve redox state. Int. J. Mol. Sci..

[34] Sen S., Domingues C.C., Rouphael C., Chou C., Kim C., Yadava N. (2015). Genetic modification of human mesenchymal stem cells helps to reduce adiposity and improve glucose tolerance in an obese diabetic mouse model. Stem Cell Res. Ther..

[35] Bolli R. (2000). The late phase of preconditioning. Circ. Res..

[36] Naderi-Meshkin H., Bahrami A.R., Bidkhori H.R., Mirahmadi M., Ahmadiankia N. (2015). Strategies to improve homing of mesenchymal stem cells for greater efficacy in stem cell therapy. Cell Biol. Int..

[37] Carrière A., Ebrahimian T.G., Dehez S., Augé N., Joffre C., André M., Arnal S., Duriez M., Barreau C., Arnaud E. (2009). Preconditioning by mitochondrial reactive oxygen species improves the proangiogenic potential of adipose-derived cells-based therapy. Arterioscler. Thromb. Vasc. Biol..

[38] Valdivia A., Pérez Y., Dominguez A., Caballero J., Hernández Y., Villalonga R. (2006). Improved pharmacological properties for superoxide dismutase modified with mannan. Biotechnol. Appl. Biochem..

[39] McCord J.M., Edeas M.A. (2005). SOD, oxidative stress and human pathologies: A brief history and a future vision. Biomed. Pharmacother..

[40] Zanetti M., Sato J., Katusic Z.S., O′Brien T. (2001). Gene transfer of superoxide dismutase isoforms reverses endothelial dysfunction in diabetic rabbit aorta. Am. J. Physiol. Heart Circ. Physiol..

[41] Khalil A.A., Jameson M.J., Broaddus W.C., Lin P.S., Dever S.M., Golding S.E., Rosenberg E., Valerie K., Chung T.D. (2013). The influence of hypoxia and pH on bioluminescence imaging of luciferase-transfected tumor cells and xenografts. Int. J. Mol. Imaging.

[42] Bertera S., Crawford M.L., Alexander A.M., Papworth G.D., Watkins S.C., Robbins P.D., Trucco M. (2003). Gene transfer of manganese superoxide dismutase extends islet graft function in a mouse model of autoimmune diabetes. Diabetes.

[43] Marrotte E.J., Chen D.D., Hakim J.S., Chen A.F. (2010). Manganese superoxide dismutase expression in endothelial progenitor cells accelerates wound healing in diabetic mice. J. Clin. Investig..

[44] Di Rocco G., Gentile A., Antonini A., Ceradini F., Wu J., Capogrossi M., Toietta G. Enhanced healing of diabetic wounds by topical administration of adipose tissue-derived stromal cells overexpressing stromal-derived factor-1: Biodistribution and engraftment analysis by bioluminescent imaging. Stem Cells Int.

[45] Wu D., Yotnda P. (2011). Induction and testing of hypoxia in cell culture. J. Vis. Exp..

[46] Di Carlo A., de Mori R., Martelli F., Pompilio G., Capogrossi M.C., Germani A. (2004). Hypoxia inhibits myogenic differentiation through accelerated myod degradation. J. Biol. Chem..

[47] Palazzotti B., Pani G., Colavitti R., De Leo M.E., Bedogni B., Borrello S., Galeotti T. (1999). Increased growth capacity of cervical-carcinoma cells over-expressing manganous superoxide dismutase. Int. J. Cancer.

[48] Di Rocco G., Gentile A., Antonini A., Truffa S., Piaggio G., Capogrossi M.C., Toietta G. (2012). Analysis of biodistribution and engraftment into the liver of genetically modified mesenchymal stromal cells derived from adipose tissue. Cell Transplant..

[49] Tiscornia G., Singer O., Verma I.M. (2006). Production and purification of lentiviral vectors. Nat. Protoc..

[50] Bedogni B., Pani G., Colavitti R., Riccio A., Borrello S., Murphy M., Smith R., Eboli M.L., Galeotti T. (2003). Redox regulation of camp-responsive element-binding protein and induction of manganous superoxide dismutase in nerve growth factor-dependent cell survival. J. Biol. Chem..

